# The Jump Shot Performance in Youth Basketball: A Systematic Review

**DOI:** 10.3390/ijerph18063283

**Published:** 2021-03-22

**Authors:** Cíntia França, Beatriz B. Gomes, Élvio Rúbio Gouveia, Andreas Ihle, Manuel J. Coelho-E-Silva

**Affiliations:** 1 Faculty of Sport Sciences and Physical Education, CIDAF, University of Coimbra, 3040-248 Coimbra, Portugal; beatrizgomes@fcdef.uc.pt (B.B.G.); mjcesilva@fcdef.uc.pt (M.J.C.-E.-S.); 2 LARSYS, Interactive Technologies Institute, 9020-105 Funchal, Portugal; erubiog@uma.pt; 3 Department of Physical Education and Sport, University of Madeira, 9020-105 Funchal, Portugal; 4 Center for the Interdisciplinary Study of Gerontology and Vulnerability, University of Geneva, 1205 Geneva, Switzerland; andreas.ihle@unige.ch; 5 Department of Psychology, University of Geneva, 1205 Geneva, Switzerland; 6 Swiss National Centre of Competence in Research LIVES—Overcoming Vulnerability: Life Course Perspectives, 1015 Lausanne, Switzerland

**Keywords:** youth players, game-related conditions, motor action, kinematics

## Abstract

Background: The basketball jump shot (JS) is consensually considered as a high-complexity specific motor skill, with a complex teaching and learning processes involved. The aim of this paper was to conduct a systematic review of the literature on the JS performance among youth basketball players. Methods: The data search was made according to Preferred Reporting Items for Systematic Reviews and Meta-Analyses (PRISMA) in the Institute for Scientific Information (ISI) Web of Knowledge, PubMed, Scopus and Sportdiscus databases until March 2021. Results: The results suggest that JS performance of youth basketball players is influenced by (i) distance to the basket, (ii) fatigue, (iii) presence of a defender and (iv) visual information available. Conclusion: This research emphasizes the crucial need for players and coaches to promote training situations matching the game reality to develop successful shooting performance of youth basketball players.

## 1. Introduction

In basketball, shooting efficacy is consensually considered by athletes and coaches as an essential component to achieve a successful performance [1,2]. The basketball shooting literature supports a reference movement pattern for the jump shot (JS) performance [3], which has emerged from biomechanics’ fundamental principles. However, even in experienced players, it is possible to observe different shooting styles with a similar efficacy percentage [1]. Therefore, inter-individual differences in the motor action’s performance could be found not only due to individual characteristics, such as anthropometry and physical capacities [4], but also due to game-related conditions [5,6,7].

Meanwhile, intra-individual differences have also been pointed out, mainly through comparing the motor action used by the same player across several shooting conditions [5]. Literature suggests that players are able to adapt their performance according to game-related conditions, such as distance variation to the basket or the presence of a defender [4,7,8]. If even in expert players, these inter- and intra-individual differences could be easily observed, it seems crucial to understand what happens in youth basketball. Being recognized as a complex specific motor skill [4,6,8], the teaching and learning process involved in JS are also complex, particularly among the more inexperienced players such as youngsters.

Overall, basketball shooting has already attracted empirical research, mainly focused on the final product: percentage of efficacy [9,10,11,12]. Indeed, efficacy could be considered the best single dependent variable of the shooting action, since it represents the game’s scoring system. However, the ratio obtained from the scored points and the total number of attempts during a game or training session fails to provide adequate feedback about the process for the shooter’s improvement, especially for the more inexperienced players. The literature claims details regarding the dynamics of motor action. For that reason, the use of a kinematic analysis emerged as crucially relevant for understanding the movement pattern, the ball trajectory and the final outcome [8,13,14].

The ball trajectory, defined by the angle, velocity and height at the moment of ball release [8], intermediates the players’ muscle action and the final outcome. Additionally, parameters of the shooter have also been studied, such as the position of the center of mass (CoM) [7,13,15]; trunk inclination [7,13]; jumping flight time [2,7,9,16]; changes in landmarks placed at joints to assess the respective position and time variations [10,11,16]. The kinematic parameters have been previously used to evaluate the game-related conditions as a source of inter and intra-individual variability among basketball shooters [7,8,10,11,14,17]. However, most of the data available are concerned with adult and experienced players.

Therefore, this paper aims to review the existent literature for the basketball JS performance, exclusively among youth basketball players. Of particular interest was to (1) determine which game-related conditions are relevant for the jump-shooting performance; (2) to understand the influence of those game-related conditions on the shooting motor action.

## 2. Methods

### 2.1. Search Strategy

A systematic review of the literature on the basketball JS was conducted according to Preferred Reporting Items for Systematic Reviews and Meta-Analyses (PRISMA) guidelines [18] for relevant publications from the last 20 years. This research was not registered on PROSPERO platform. To ensure the quality of the articles selected, the data search was conducted in the Institute for Scientific Information (ISI) Web of Knowledge, PubMed, Scopus and Sportdiscus databases until March 2021. The main term used in the research process was “basketball shot”, associated with the following keywords: “performance”, “ball trajectory” and “young players”. The Boolean phrase used in the search was—AND—to identify the largest number of documents possible that complied with the established conditions. The Boolean phrase OR has not been used, since it was intended to achieve exclusive information in the search process. For the documents to be included and analyzed, they had to fulfil the defined inclusion and exclusion criteria. Two authors individually performed the search using the strategy previously mentioned. The same authors screened the citations and abstracts to identify documents that could be included in the review. In case of potential doubt about the selection of one particular article, both authors analyzed the full article to determine whether the inclusion criteria were met. In the case of disagreements between the two authors in terms of the inclusion criteria, a third author analyzed the full article and made the final decision.

### 2.2. Eligibility Criteria

The inclusion criteria for these articles were (i) data including one or more variables that affect the JS performance; (ii) sample uniquely composed by basketball players; (iii) participants (boys and/or girls) aged between 10 and 19 years; (iv) presented quantitative or theoretical data; (v) written in English, Spanish or Portuguese languages; (vi) published in the past 20 years (2000–2020). Manuscripts were excluded if they did not refer to the JS performance; were only focused on the free-throw motor action; were developed in Paralympic basketball or school samples not engaged in competitive and organized youth basketball; papers developed in high-level competitions such as Euroleague, National Basketball Association (NBA) or Olympic games; conference papers or other material that cannot be referenced.

## 3. Results

**Figure 1:**
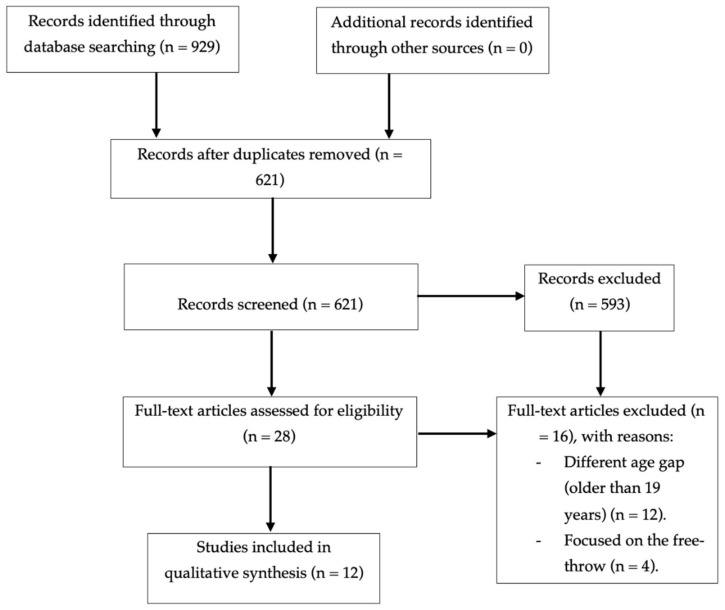
Flow chart of the document’s selection process.

A total of 929 studies were selected, as presented in Figure 1. References were adequately managed (Zotero 5.0.94), and duplicates were automatically deleted (n = 308). The 621 articles remaining were examined for relevance based on title and keywords. Additionally, conference papers, items that could not be referenced and documents classified as not scientific relevant were also eliminated (n = 593). Articles that did not perform some type of analysis of the JS performance were considered not relevant (e.g., papers developed on Paralympic basketball; in high-level competitions such as Euroleague, NBA or Olympic games; injuries assessment or prevention; physical fitness; game-analysis performance; nutritional aspects; coaching). Thus, 28 articles have remained for full-text assessment remaining. From those, papers developed with elite players older than 19 years (n = 12) and papers focused on the free-throw analysis (n = 4) were also excluded. Thus, a final selection of 12 papers has been used for this review.

### 3.1. Evidence Synthesis

Twelve documents were selected for this analysis (Table 1). Evidence was synthesized according to the following variables: (i) distance variation to the basket (four studies) [16,19,20,21]; (ii) fatigue (four studies) [15,22,23,24]; (iii) presence of a defender (two studies) [9,25]; (iv) visual information available (two studies) [12,26]. One of the documents has reported data on the JS performance both on a defender’s presence and on the players’ gaze behavior (visual information) [25]. Besides, only two studies have used exclusively female participants, eight studies used male participants, and two studies used participants both boys and girls. In nine studies, participants were adolescent players. From the total sample, seven papers have presented results of the kinematic parameters.

### 3.2. Main Findings

#### 3.2.1. Distance to the Basket

Regarding distance to the basket, the first study among six girls aged between 10 and 11 years considered a two-dimension kinematic analysis used to analyze the effects of five shooting distances to the basket (2, 3, 4, 5 and 6 m) on the JS performance [19]. Authors have focused their attention on the angles formed by the shoulder, elbow and knee joint. Results showed lower values for all the variables previously mentioned with the increase in the shooting distance. The ball release variables were not assessed. However, the authors pointed out a high variability of the movement pattern as the distance to the basket was increased [19].

In another study, using a two-dimensional kinematic analysis, authors have examined the JS performed by boys aged 12.1 ± 1.4 years among three distances to the basket (2.8, 4.6 and 6.4 m) [20]. Significant differences were found when the performance at 2.8 m was compared with the performance at 6.4 m for the ball release parameters: height of release (M = 1.93 ± 0.07 m; M = 1.79 ± 0.05 m) (*p* < 0.01); angle of release (M = 68.7 ± 3.2°; M = 57.8 ± 3.4°) (*p* < 0.05); velocity of release (M = 5.43 ± 0.1 m/s; M = 7.37 ± 0.2 m/s) (*p* < 0.01). The maximum angular velocities of the elbow and the shoulder joints have progressively increased, with a statistically significant value (*p* < 0.05), due to the distance increase. The mean value for the elbow joint angular velocity was 637.2 ± 39.7°/s at 2.8 m condition and 855.3 ± 40.4°/s at 6.4 m condition. On the other hand, the mean value for the shoulder joint angular velocity was 422.3 ± 38.7°/s at 2.8 m condition and 576.5 ± 50.2°/s at 6.4 m condition. Meanwhile, at ball release, the mean value for the elbow joint angular velocity was superior at 4.6 m (M = 599.6 ± 42.6°/s) when compared to the 2.8 m condition (M = 509.0 ± 48.6°/s), but lower when compared to the 6.4 m condition (M = 593.6 ± 64.9°/s). Simultaneously, the mean values for the shoulder joint angular velocity at ball release have increased from the shorter to the longer shooting distance, although with no significant statistical impact. Additionally, significant differences were detected in the body’s horizontal displacement (*p* < 0.05) when the shots at 2.8 m were compared with the shots at 6.4 m condition, 0.35 ± 0.02 m and 0.49 ± 0.03 m, respectively [20].

In a three-dimensional kinematic analysis, the joints’ angular velocities were assessed during the JS performance in a group of 14 top-level male players aged 15.4 ± 0.5 years [16]. The authors aimed to describe the joint angular velocities during the JS performed three distances to the basket (3.75, 5.75 and 6.75 m). The elbow joint’s maximum angular velocities increased significantly (*p* < 0.05) when the shooting range was longer, with mean values of 923.4 ± 86.4°/s and 1212.4 ± 158.8°/s, at 3.75 and 6.75 m, respectively. The mean values for the shoulder joint were 444.2 ± 78.9°/s at 3.75 m condition and 718.6 ± 174.2°/s at 6.75 m condition, illustrating significant differences in the maximum angular velocities for this joint as well. The angular velocity of the elbow and the shoulder joint at ball release was also higher at longer shooting distances. On the contrary, no significant differences were reported on the wrist joint’s maximum angular velocities, with mean values of 1528 ± 383.2°/s at 3.75 m and 1731 ± 658.5°/s at 6.75 m [16].

In another study, through a three-dimensional kinematic analysis, the CoM’s behavior, the player’s shooting speed, the entry angle of the ball when approaching the rim and the shoulder angle at release were observed in 2 and 3-point shots performed by 48 players U16 and U18 male and female categories who participated on the European Championship in 2017 [21]. In all categories (male and female), significant differences were found between 2- and 3-point shot on the entry angle of the ball (*p* < 0.01) and on the player’s shooting speed (*p* < 0.01). In general, the 2-point shots presented lower entry angles of the ball and were performed faster in comparison to the 3-point shots. Across the same shooting conditions, results also showed that males shot with a higher CoM’s difference in the vertical direction, with a higher release shoulder angle and with a higher entry angle of the ball, when compared to female players. Finally, the efficacy was lower in the 3-point shot and females showed lower shooting percentages of efficacy than male, except the 2-point shot at U18 category [21].

#### 3.2.2. Fatigue

Three studies have examined fatigue through specific protocols and used maximal heart rate (HR) percentages as an indicator of physical fatigue [15,22,23]. Significant differences were found on the 3-point percentage of efficacy in a group of 24 young male basketball players aged 16.3 ± 0.6, at higher rates of maximal HR. When comparing three conditions of HR measurements, respectively 0, 50 and 80%HR, the authors observed a progressive decrease in efficacy as a response to fatigue increase [22]. Although with no significant differences (*p* = 0.25), a 15% reduction in shooting efficacy rate at 50%HR was observed when compared to the rest condition (0%HR). On the other hand, 3-point efficacy has decreased significantly 28% when shooting at 80%HR was compared to the rest condition (0%HR) (*p* < 0.05) [22]. Another study applied a similar protocol of maximal HR measurement in 22 young male basketball players aged 15.7 ± 0.9 years to evaluate the effect of fatigue on the 2-point shot efficacy [23]. Results showed lower percentages of efficacy at 80%HR when compared to 50%HR (−21%) and to 0%HR (−29%) (*p* < 0.01). No differences in shooting efficacy were found when the performance at 50%HR was compared to the rest condition (0%HR) (*p* = 0.34) [23].

Meanwhile, a kinematic analysis aimed to investigate fatigue’s effects on the 3-point shot performed by ten elite male basketball players aged 16.3 ± 1.2 years [15]. At 88%HR, no significant differences were found in the ball release parameters (release velocity *p* = 0.80, release angle *p* = 0.14 and release height *p* = 0.51) when the non-fatigued was compared to the fatigued condition. In addition, the angles formed by the shoulder, elbow, wrist, hip, knee and ankle joint did not show significant statistical differences between shooting conditions [15].

Another study has evaluated several kinematic parameters in fatiguing conditions. Instead of the HR measurement, the level of blood lactate concentration was used as a fatigue indicator [24]. The maximum angular velocities of the shoulder and wrist joint were significantly scaling down with the greater manifestation of fatigue. In the first shooting series, the shoulder’s mean maximum angular velocity was 510.89 ± 22.10°/s and in the final series was 484.46 ± 18.56°/s (*p* = 0.02). The wrist’s mean maximum angular velocity has also decreased significantly (*p* < 0.01) from the first (1227.02 ± 143.73°/s) to the last series (950.04 ± 53.23°/s). Moreover, the height of ball release was also significantly lower (*p* < 0.01) in the fatigued condition (2.47 ± 0.02 m) when compared with the first shooting series (2.58 ± 0.02 m). No differences were reported on the angle and velocity at the moment of ball release.

#### 3.2.3. Presence of a Defender

Two studies have assessed the influence of a defender on the JS [9,25]. In 12 highly skilled male basketball players aged 17.8 ± 1.1 years, the influence of a defender was examined on the performance of several types of shooting [9]. Results showed that contested shots (M = 0.99 s) were performed significantly faster (*p* < 0.01) than uncontested shots (M = 1.08 s). Besides, the jump time was significantly longer (*p* < 0.01) in defended conditions (M = 0.43 s) compared to that observed in the undefended conditions (M = 0.40 s). The ball flight time was also longer in defended conditions (M = 0.96 s) than in undefended conditions (M = 0.83 s). Regarding efficacy, the percentage was significantly lower (*p* < 0.01) in defended conditions (M = 41.1%) compared to undefended conditions (M = 63.9%) [9].

In the second study, the effect of the presence of a defender on jump-shooting was evaluated among 13 talented young female basketball players aged 16.8 ± 1.8 years. Authors have assessed the percentage of efficacy, the ball’s trajectory, the total time of shot execution and the gaze behavior during the shot performance as well [25]. Results showed that the ball trajectory was longer in contested (M = 1027 ± 69 ms) than in uncontested shots (M = 994 ± 55 ms) (*p* < 0.01). Contested shots were also performed faster (M = 817 ± 82 ms) than uncontested shots (M = 896 ± 100 ms) (*p* < 0.01). Moreover, the percentage of efficacy was almost similar between both conditions, with 52.2% efficacy in the contested shots and 51.3% of efficacy in the uncontested ones (*p* = 0.85) [25]. On the other hand, the gaze behavior was studied using eye-tracking glasses and video recording to compare the duration and the onset of the final fixation on the rim before the ball release. In this particular study, a “fixation” was defined as a gaze maintained on a specified location (e.g., the rim) for a period equal or superior to 100 ms or three sequential frames. Overall, the final fixation on the rim was shorter and occurred later on contested (M = 364 ± 191 ms) than in uncontested condition (M = 443 ± 221 ms) (*p* = 0.39). Furthermore, the authors also reported differences in the gaze behavior between the highly skilled players and their counterparts. While the highly skilled players’ gaze behavior was not affected by the defender’s presence, players who shot with less efficacy with a defender also presented shorter final fixations on the rim than their highly skilled peers [25].

#### 3.2.4. Visual Information

Regarding visual information available, the first study in 17 male participants aged 18.8 ± 0.6 years composed of ten intermediately skilled and seven highly skilled basketball players, the “quiet-eye” (QE) functionality was compared between a defended and undefended condition [26]. Authors reported that successful shots performed by highly skilled players in defended game situations were associated with longer QE durations (M = 452 ± 43.3 ms). In comparison, unsuccessful shots were related to shorter quiet eye duration (M = 349 ± 54.4 ms). The same results were found for the intermediate-skilled players, with longer QE durations being found for scored shots (M = 431.9 ± 36.8 ms) compared with missed shots (322.8 ± 47.3 ms) in defended condition. As expected, the shooting performance was significantly higher in the undefended (M = 65.6 ± 16.1%) than in defended situations (M = 44.5 ± 14.2%) (*p* < 0.01) [26].

The second study was based on an intervention with goggle training between six adolescent male basketball players aged 17 years. Participants were submitted to 8 weeks of visual control training where they only had a vision during the final 350 ms before the moment of ball release [12]. Besides, players had screen training sessions, where they had to shoot behind a screen where they could barely see the top of the small rectangle on the backboard. This intervention aimed to manipulate the vision so that participants could only see the basket during the final instances before ball release. Results showed that participants submitted to the training sessions had extended the last fixation duration when shooting with late vision. This suggests an increase in their ability to pick up relevant information during the final instance before ball release. Moreover, the shooting percentages were compared between the pre-intervention period (M = 46.1 ± 10.2%) and post-intervention period (M = 60.6 ± 12.1%), showing an improvement of almost 15% of the game percentage of efficacy after the intervention (*p* < 0.05). On the contrary, the control group’s efficacy was maintained practically constant, with 42.5 ± 3.7% on the pre-intervention period and 42.2 ± 3.6% on the post-intervention (*p* < 0.10) [12].

## 4. Discussion

This paper aimed to identify which game-related conditions could influence the JS performed by youth basketball players and describe their effect on the motor action used. Our detailed analysis of the empirical research suggests that the JS is influenced by (i) distance to the basket, (ii) fatigue, (iii) presence of a defender and (iv) visual information available.

Shooting efficiency and efficacy have been considered by coaches and basketball players, substantially affected by distance to the basket [3]. Several authors have investigated the influence of the shooting distance on the JS performance by experienced players through two- and three-dimensional kinematic analyses [8,10,11,14]. Consensually, literature mentions lower angles and higher velocities at the moment of ball release when the distance to the basket is increased [6]. In our research, only one study evaluated the ball release variables when the shooting distance was increased [20]. The inverse relationship between the angle and velocity mentioned on literature was also reported in boys aged 12.1 ± 1.4 years [20]. However, it seems essential to develop more studies related to the ball release variables (height, angle and velocity) mainly to compare the variation of those variables between adults and youngsters. Due to their accumulated experience, adult players should be more able to coordinate the motor action and to accomplish a successful shot.

Additionally, basketball-shooting literature mentions higher maximum angular velocities of the shoulder and elbow joints in longer shots [8,11,14]. This conclusion is in line with the results reported on youth basketball players, both in top-level male players aged 15.4 ± 0.5 years [16] and intermediate-skilled boys aged 12.1 ± 1.4 years [20]. For the elbow and the shoulder joints, an increase of almost 300°/s in the maximum angular velocities was found in a group of 14 top-level U16 male players when the JS performance at 3.75 m was compared to 6.75 m [16]. There was also an increase in the elbow’s and shoulder’s angular velocity in a younger population when the performance at a shorter distance (2.8 m) was compared with the performance at a longer distance (6.4 m). Indeed, at longer distances to the basket, players need to adapt their body segments to generate a greater impulse to shoot, since the ball’s trajectory will be longer [4]. Thus, joints’ angular velocities tend to increase, and the angles formed by the body’s segments tend to decrease as a way to create an appropriate impulse to shoot [4,14,19].

Meanwhile, in boys, significant differences were also detected in the body’s horizontal displacement when the performance at a shorter distance (M = 0.35 ± 0.02 m) was compared with the longer distance (M = 0.49 ± 0.03 m) [20]. The horizontal displacement seems to be accentuated by the increased distance to the basket. However, highly skilled shooters have been observed to have a less horizontal shift in their CoM than their less-skilled peers [6]. Thus, it should be expected that a more significant horizontal displacement may be observed in inexperienced performers as youngsters, mainly due to the higher impulse created to shoot when the distance to the basket is increased.

The angles formed by the joints have also been used on the assessment of the shooting motor action. In girls aged 10–11 years, authors reported the decrease in the mean values of the angles formed by the elbow, shoulder and knee joint when the performance at a shorter distance (2 m) was compared with the performance at a longer distance (6 m) [19]. In fact, in the knee joint’s particular case, the minimum knee flexion value is a good indicator of the squat movement performed during the shot’s preparatory phase, which will have a significant repercussion on the jump phase. Therefore, it is expected that lower values of knee flexion should be observed when players’ aim to jump more to achieve longer ball trajectories.

In another study with U16 and U18 male and female players, differences between 2- and 3-point shots were reported on the entry angle of the ball when approaching the rim and on player’s shooting speed [21]. The 2-point shots presented lower entry angles of the ball and were performed faster. The entry angle of the ball represents a main criterion for successful performance, since as the entry angle increases, the width of the basket increases as well [4,14]. In this study, the authors did not report data concerning the ball release parameters (angle, velocity and height). However, previous studies have shown that the ball release angle tends to decrease at longer shooting distances [6,8,11,14]. For that reason, it would be expected that lower entry angles of the ball in the rim were reported in 3-point shots when compared with the 2-point shot, since greater values of the angle of release should generate greater values of the entry angle as well [4]. Players showed lower shoulder angles at ball release in the 2-point shot performance, which should explain the lower entry angles of the ball. On the other hand, the need to create a higher impulse to shoot from longer distances justifies the faster performance of the 2-points shots when compared with the 3-point shots.

In sports literature, fatigue has been recognized as an adverse variable for game performance [17]. In youth basketball, the 2-point and the 3-point performance in fatigued and non-fatigued conditions were examined through the percentage of efficacy [22,23]. The maximal HR measurement was used as a fatigue indicator. In both situations, the most significant decrease in the shot’s success was reported at 80%HR when compared with a rest condition (0%HR). Although there was a decrease in the efficacy rate at 50%HR when compared to the rest condition (0%HR), it did not significantly impact the performance. Considering these results, it seems that efficacy is only seriously compromised at higher fatigued conditions. However, the percentage of efficacy is still not enough to comprehend the motor action, since it only represents the shot’s output.

Certainly, fatigue is widely considered a modifier of the kinematic of various sports-related movements [15,17]. Only two studies on the JS were found on young basketball players on the JS performance’s kinematic assessment in fatigued conditions. A case-study developed with a member of the Croatian U18 Men’s National Team showed a significant decrease in the mean values of the maximum angular velocities of the shoulder and wrist joints in fatigued conditions in 3-point shots performance [24]. On the other hand, the maximum angular velocities of the lower extremities increased in a fatigued state. The height of ball release was the only release parameter that showed significant differences across conditions, with lower values being observed in a fatigued state [24]. Although these data are concerned to only one individual, it could be assumed that even with the reorganization of the body’s behavior in response to accumulated fatigue, the ball release parameters stayed mostly unaffected.

Meanwhile, after a protocol of repeated sprints and jumps, the 3-point shot was evaluated in a group of ten elite male basketball players aged 16.3 ± 1.2 years [15]. At 88% of the maximal HR, no significant differences were detected in the joint angle’s movements or the ball release parameters when the non-fatigued condition was compared to the fatigued state. Unfortunately, no percentages of efficacy were reported in both situations. However, it could be assumed that efficacy was not seriously compromised by fatigue, since the performance of the motor action did not differ significantly. It must be referenced that only elite youth players were involved in this study, who seem to be able to cope with physical fatigue while performing coordinated movements such as the 3-point shot [15]. The lack of studies and the different backgrounds of the players and methods used to assess fatigue demand additional research on the topic, since some of the results founds are contrary. Once fatigue has been recognized as an adverse variable for game performance, more information on the subject brings greater input for the organization of training sessions.

Indeed, elite players should be more capable of performing a coherent motor action even through several game-related conditions, including the presence of a defender or restricted visual information available. The players’ field of view is often affected by defenders’ and teammates’ positioning, representing a dynamic system [26]. In precision tasks, such as basketball shooting, a gaze behavior labelled QE has been found to explain differences in motor expertise. The QE is defined as a fixation or tracking gaze located on a specific object or location in the environment [26,27,28,29]. The presence of a defender while shooting represents a severe constraint for the JS performance, mostly constituting an obstacle for the target (basket) visualization. In youngsters, authors described the contested shots as faster, with long jump phases and longer ball trajectories [9,25]. This behavior has also been observed in adult expert players [7] and should be related to the shooter’s strategy to avoid the defender’s interception [7,9].

On the other hand, other studies have mentioned that expert players are able to extract relevant information earlier than their less-skilled peers [28]. This allows them to select and execute appropriate motor responses more accurately [26,27]. In fact, successful jump-shooting performance has been associated with the players’ ability to pick up relevant information during the final instance before ball release [27,28,29]. After being submitted to eight weeks of shooting with late vision training, six adolescent male basketball players aged 17 years have extended the final fixation duration during the jump-shooting [12]. In addition, an improvement of efficacy percentages during the competition was also observed in comparison to the control group. Authors have found that visual control training can change the temporal pattern of shooting and improve performance by enhancing information detection timing [12].

Similar results were reported in highly-skilled youth players, who did not show significant differences in the gaze behavior or the percentage of efficacy when shooting against a defender. On the contrary, intermediate-skilled players shot worst with a defender (less efficacy) and presented shorter final fixations on the rim than their highly skilled counterparts [25]. This result all together brings important practical implication on the training process, since the position on the court of the teammates and defenders during gameplay is extremely dynamic, which will impact the shooter’s field of view.

To the best of our knowledge, this is the first study developed to explore the JS motor action’s performance in the early stages of basketball’s long-term development. Most of the previous studies have so far been focused on expert or adolescent highly skilled participants. Moreover, the percentage of efficacy has been widely used as the primary indicator of shooting success, which fails to provide adequate feedback for the shooter’s improvement. Thus, the kinematic variables’ analysis emerges as fundamentally relevant to understand the performance adjustments needed according to game-related conditions. The few studies eligible for this review represent a limitation. However, aggregating all available empirical work on this topic represents a strength of this paper. It emphasizes the need for future investigations on this topic. Since the early stages of basketball’s long-term development are crucial to the specific motor skills acquisition and, consequently, for players to achieve successful performance at elite levels, it is fundamental to understand the best strategies for skill improvement already at younger ages. Therefore, future research should be developed in youth, particularly with a focus on the comprehension of the motor action, which could be achieved through the assessment of the kinematic parameters.

## 5. Conclusions

Our detailed analysis of the body of empirical evidence suggests that in youth basketball, the JS performance is influenced by distance to the basket, accumulated fatigue, presence of a defender and visual information available. There are intra- and inter-individual differences in the motor action performance due to players’ characteristics, such as previous sports experience and as a response to game-related conditions. Critical practical implications for players and coaches have emerged from this review, in particular the need to promote dynamic shooting training situations that should be matching the game reality.

## Figures and Tables

**Table 1:** Studies description and identification of the variables considered.

Author	Aim(s) of the Study	Participants	Game-Related Conditions Considered	Main Results
Vencurik et al. [21]	To compare the kinematic variables of 2- and 3-point shot.	48 male and female basketball players U16 and U18	Distance variation to the basket	2-point shots showed lower entry angles of the ball and were performed faster than the 3-point shots.
Rupcic et al. [24]	To evaluate the effect of fatigue on the kinematic parameters of the 2- and 3-point JS.	1 male basketball player aged 17 years	Fatigue	Height of ball release decreased and angular velocities of joints on the upper extremity decreased in fatigued conditions.
Van Maarseveen & Oudejans [25]	Effects of a defender contesting the JS on the performance and gaze behavior.	13 female basketball players aged 16.8 ± 1.8 years	Presence of a defender and visual information	Contested shots were performed faster, had a long jump phase and ball flight and presented shorter final fixations than uncontested shots.
Slawinski et al. [15]	Impact of physical fatigue on upper and lower limb joint kinematics and in ball release parameters	10 elite basketball players, six male and four female, aged 16.3 ± 1.2 years	Fatigue	No differences were found for ball release variables, centre of mass vertical displacement and jump height, between fatigue and non-fatigued conditions.
Ardigo et al. [22]	Effects of a fatigue protocol on the 3-point shooting efficacy.	24 male basketball players aged 16.3 ± 0.6 years	Fatigue	80%HRMax had a significantly negative influence on the 3-point shooting percentage of efficacy.
Padulo et al. [23]	Effects of a fatigue protocol on the 2-point shooting efficacy.	22 male basketball players aged 15.7 ± 0.9 years	Fatigue	80%HRMax had a significantly negative influence on the 2-point shooting percentage of efficacy.
Podmenik et al. [16]	To describe joint angular velocities during the performance with the increase in the shooting distance.	14 top-level male basketball players aged 15.4 ± 0.5 years	Distance variation to the basket	Maximum angular velocities were generally similar at shorter distances but were higher at a longer distance.
Klostermann et al. [26]	To explore the quiet eye (QE) functionality in a defended and undefended condition.	17 male basketball players aged 18.8 ± 0.6 years	Visual information	Successful shots were associated with longer QE durations.
Gorman & Maloney [9]	Examine the influence of a defender on the performance of a basketball shot using five different shot types.	12 male basketball players aged 17.8 ± 1.1 years	Presence of a defender	Presence of a defender led to faster shot execution times, longer jump times and to an increase in the ball flight time.
Okazaki et al. [20]	Analyze the effect of the increase in the shooting distance on the JS performed by boys.	15 boys aged 12.1 ± 1.4.	Distance variation to the basket	Significant differences on the ball release variables at longer shooting distances; maximum angular velocities of the shoulder and elbow joints were greater when the distance was increased.
González-Fimbres et al. [19]	Impact of the increase in the shooting distance on the JS kinematics performed by girls.	6 girls aged between 10–11 years.	Distance variation to the basket	Angles formed by the body’s joints had increased when the shooting distance was increased.
Oudejans [12]	Effects of perceptual training on basketball JS percentage of efficacy.	10 adolescent male basketball players aged 17 years	Visual information	Visual training has increased players’ ability to pick up relevant information during the final instance before ball release, with an effect on their percentage of efficacy.

## Data Availability

The data presented in this study are available on request from the corresponding author.
